# The Betrayal Aversion Elicitation Task: An Individual Level Betrayal Aversion Measure

**DOI:** 10.1371/journal.pone.0137491

**Published:** 2015-09-02

**Authors:** Jason Aimone, Sheryl Ball, Brooks King-Casas

**Affiliations:** 1 Department of Economics, Hankamer School of Business, Baylor University, Waco, Texas, United States of America; 2 Virginia Tech Carilion Research Institute, Virginia Tech, Roanoke, Virginia, United States of America; 3 Department of Economics, Virginia Tech, Blacksburg, Virginia, United States of America; 4 Department of Psychology, Virginia Tech, Blacksburg, Virginia, United States of America; 5 Research Service Line, Salem Veterans Affairs Medical Center, Salem, Virginia, United States of America; 6 Department of Psychiatry, Virginia Tech Carilion School of Medicine, Roanoke, Virginia, United States of America; 7 VT-WFU School of Biomedical Engineering and Sciences, Blacksburg, Virginia, United States of America; George Mason University, UNITED STATES

## Abstract

Research on betrayal aversion shows that individuals’ response to risk depends not only on probabilities and payoffs, but also on whether the risk includes a betrayal of trust. While previous studies focus on measuring aggregate levels of betrayal aversion, the connection between an individual’s own betrayal aversion and other individually varying factors, including risk preferences, are currently unexplored. This paper develops a new task to elicit an individual’s level of betrayal aversion that can then be compared to individual characteristics. We demonstrate the feasibility of our new task and show that our aggregate individual results are consistent with previous studies. We then use this classification to ask whether betrayal aversion is correlated with risk aversion. While we find risk aversion and betrayal aversion have no significant relationship, we do observe that risk aversion is correlated with non-social risk preferences, but not the social, betrayal related, risk component of the new task.

## Introduction

A number of studies now support the idea that aversion to betrayal is an important factor influencing how individuals approach and respond to risky outcomes [1–3]. These studies have identified the presence and influence of this factor across groups of individuals. While extant designs have been effective in demonstrating how betrayal aversion influences a population’s decisions on average, they do not allow examination of an individual’s level of betrayal aversion. For instance, no studies to date have explored whether an individual’s level of betrayal aversion is related to their risk preferences, a potentially related construct. To examine such relationships, one must first develop a procedure to elicit an individual subjects’ level of betrayal aversion, and this paper describes the first such procedure.

The current project has two central goals. The first goal, detailed in section 3, is to develop an elicitation procedure to measure betrayal aversion at the individual level. This is important because it will allow researchers to conduct studies of how betrayal aversion is related to individual decisions and preferences. While we employ our new elicitation procedure in a personal betrayal aversion setting (in both the gain and loss domains), the task could be easily modified to explore betrayal aversion by 3^rd^ parties or betrayal aversion over objects of trust and safety as well. A second goal of this research, detailed in section 4, is to use the novel elicitation procedure to examine the relationship of betrayal aversion and risk aversion. We hypothesize that risk attitudes in non-social situations will be related to risk attitudes in social situations, while neither will be related to the novel measure of betrayal aversion. Before describing our elicitation procedure and application in sections 3 and 4, we examine prior treatments of the betrayal aversion construct. Betrayal aversion has been defined and studied in different ways by psychologists, economists, and legal scholars. In section 2, we briefly review the history of betrayal aversion research and suggest that our approach to this phenomenon, which addresses issues at an individual rather than group level, should be of interest to scholars across disciplines.

## Previous Treatments of Betrayal Aversion

In an attempt to synthesize the research on betrayal aversion, we suggest that most studies share a common understanding of the phenomenon. That is, betrayal aversion is considered the strong dislike for violations of trust norms. Thus, a betrayal averse person experiences disutility from the experience, anticipation or observation of non-reciprocated trust. This disutility can be inferred from the decreased willingness to take a risky action, or increased willingness to punish following the outcome.

The prerequisite to studying betrayal aversion is an understanding of betrayal. A common example of betrayal is Judas, who reveals Jesus’s identity for his own self-interest. The betrayal violates the norms implicit in a trust relationship. For some, the act of betrayal must be a result of a person’s intent to betray for self-interest [4], not an inadvertent mistake [5]. For others, however, betrayal does not depend on human intentions at all. Betrayal aversion initially surfaced in the behavioral and consumer decision-making literature (1, henceforth, KG). Their aim was to investigate the norm that objects should not cause the harm they are meant to prevent. KG ask participants to select between identical cars with different airbags. The airbag for one car was associated with a 2% risk of dying in a serious accident, and the airbag for the other car was associated with just a 1% risk. From a strict risk minimization standpoint, everyone should choose the car associated with the 1% death risk. But participants were told that the airbag on the second car (but not the first car) carried with it a very small (0.01%) additional risk, namely, a risk that the airbag itself would come out with such force that it could cause death in an accident that a person would otherwise have survived. KG describe this additional risk as a betrayal risk because the very object that we expect to provide protection from death may effectively “betray” us by causing death. The authors reason that, while safety products such as airbags cannot explicitly make promises to protect us, we nonetheless trust them. This opens the door for feelings of betrayal when they cause the very harm they were purchased to guard against. A later paper examining emotion’s role in betrayal aversion finds that factors such as positive imagery can encourage people to pick lower risk options with a chance for betrayal over higher risk options [6]

KG also show that the level of betrayal varies substantially depending on the extent to which intentional betrayers are perceived to have a “duty to protect”. For example, KG show that participants report that betrayal is higher when a military officer commits treason than when it is committed by an orchestra conductor. Similarly, citizens are likely to feel that crimes committed by those entrusted to guard against harm, for example, a day care worker who abuses a child, should face more severe punishments (KG; [7]). When this does not occur there may be pressure for courts to respond by developing new legal doctrines [8]; for example, whether it is reasonable for courts to award punitive damages to those who cause the negative emotional outcomes a betrayal inflicts [9]. Similarly, outside of a legal context, consumers who feel betrayed by a service failure may seek to punish the involved firm through negative reviews, public complaints, or poorly treating the firm’s employees [10].

In contrast to the work on consumer and legal decision-making, a third area of research on betrayal aversion has developed in an effort to understand how people respond to betrayal risks, relative to other types of risk. This work has focused on personal betrayals in which the act of betrayal is specifically the violation of the reciprocity norm after monetary trust. This type of research involves teasing the risk and trust aspects of a trust decision apart, leaving betrayal aversion as the remaining explanatory variable. In this work, one group of participants plays a standard trust game while the other group plays a trust game where random chance, rather than a counterpart, determines the trustee’s return. One approach [2,11–13] finds participants requiring a higher minimum acceptable probability, MAP, of the reciprocal return in the trust game compared to a risky dictator game, indicating betrayal aversion. A different approach adopted by [3,14–16] measures frequencies of individuals “trusting” in a standard trust game environment and in an environment that shields investors from knowledge of personal betrayal. They observe higher trust rates in the latter treatment indicating the existence of betrayal aversion. [3] demonstrate a beneficial role of betrayal aversion in social exchange, finding that when betrayal aversion is removed from a trust environment trust rates actually plummet due to increased rates of betrayal, which the authors suggest is due to trustees knowing that they no longer cause emotional harm by betraying trust and thus are more willing to do it.

In the neuroscience literature, [17] have used similar trust and risk games to indicate a potential role for oxytocin in mitigating betrayal aversion, allowing for social approach and social trust and risk taking. Following oxytocin administration, participants were more willing to expose themselves to the risk of betrayal. On the other hand they were no more willing to expose themselves to equivalent risk generated by a random device (see [18] for a related follow up study). Similarly, Aimone and co-authors [16] used trust and risk games combined with functional magnetic resonance imaging to implicate anterior insula in betrayal aversion.

The focus on betrayal aversion stemming from aversion to personal betrayals extends to research in many other fields as well. For example, evidence [19] shows that managers of firms, who need to trust subordinates, are also are impacted by betrayal aversion, observing that principals in principal agent games would frequently spend more on implementing institutions that would completely prevent betrayals than could ever be lost from acts of betrayal by their trust agents.

While researchers have approached betrayal aversion differently across fields, the use of a between subject design is a common, as the goal is to identify conditions under which aversion is observed. However, these designs do not allow us to examine how betrayal aversion at the individual level, relates to other constructs at the individual level. In this paper, we develop a means to easily measure an individual’s level of betrayal aversion in a specific environment. We focus in this paper on aversion to a personal betrayal, though the task could be easily modified to explore impersonal betrayal aversion, such as the betrayal of third-parties in a legal context or betrayal aversion to objects of safety in a consumer context. This opens up the possibility to research a realm of different research questions at an individual level rather than a group level.

## Experimental Design and Procedure

### Ethics Statement

This research was reviewed and approved by the "Virginia Tech Institutional Review Board for Research Involving Human Subjects." This board serves as the University's ethics committee and is guided by the ethical principles described in the “Belmont Report” and in applicable federal regulations. Written informed consent using a consent process approved by the university ethics committee was received from all participants.

### Experimental Design

#### Betrayal Aversion

**Fig 1:**
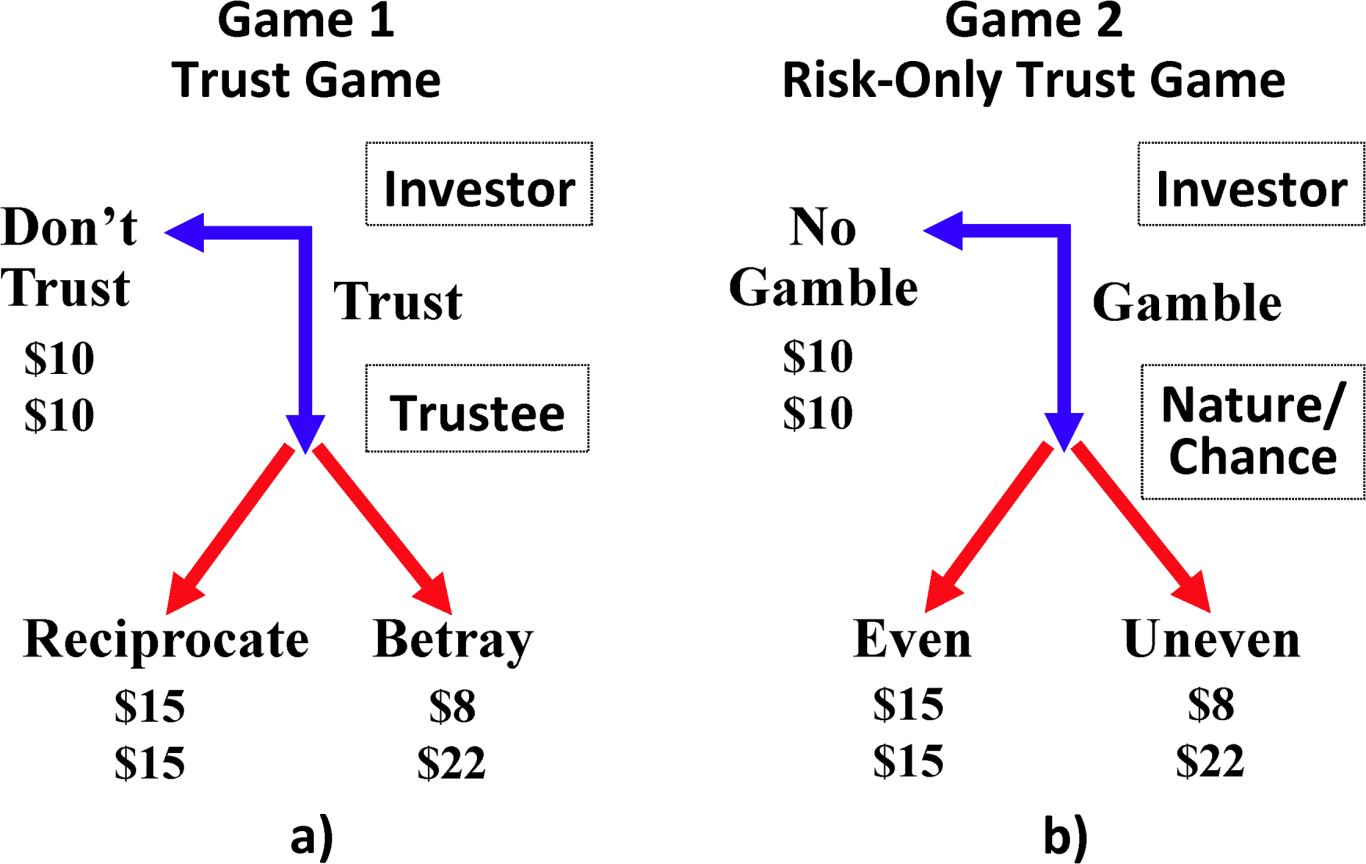
Extensive form representation of the two games in the Betrayal Aversion Elicitation Task: a) the Trust Game; b) the Risk-Only Trust Game. In the Trust game (Risk-Only Trust Game), the first move is made by the investor. If the investor chooses Don't Trust (No Gamble) the game ends and each participant earns $10, otherwise the Trustee makes a choice of Reciprocate or Betray (Nature/Chance determines the outcome). Note the probability that nature/chance chooses “Even” in Game 2 is identical to the actual probability that the investor has of being randomly matched with a trustee who chose “Reciprocate” in Game 1.

We draw elements from the betrayal aversion studies of [2] and [14] to develop a novel elicitation procedure that allows us to identify individual preferences. The Betrayal Aversion Elicitation Task (BAET) includes two games. Game 1 is a binary one-shot trust game (Fig 1A). Game 2 is a risk-only game which retains the structure and payoffs of the one-shot trust game, but replaces the second stage decision made by the trustee with a random decision (Fig 1B).

The BAET has three steps. Participants are first asked to report which move they would choose in the role of trustee in Game 1 if their investor counterpart chooses to trust. These decisions determine the actual probability, P_-i_, that each participant, i, has of being randomly assigned to a counterpart,-i, who chooses to reciprocate trust in the next two steps of the task. While step one does not provide data about an individual’s level of betrayal aversion, it provides necessary data for steps two and three.

In the second step, the trust game, participants are asked what they would do in the role of the investor in Game 1. Each participant i reports the minimum acceptable probability for which he or she chooses to trust (MAP_ti_). If P_-i_ > MAP_ti_, then the participant’s outcome is determined using “trust” as the investor strategy and the choice of a randomly selected opponent as the trustee strategy, otherwise the investor ends the game by selecting “don’t trust”. Rather than asking for an MAP directly (as in [2]), we use the strategy method for nineteen different possible values of P-i. Alternatively, researchers adopting the BAET could expand or contract the number of possible values of P-i, or even adopt a free response method, to meet their research needs.

In the final step, the risky dictator game, participants are asked what they would do in the role of investor in Game 2. In this game the trustee’s strategy is a lottery where the probability of an even split P_-i_, determines the probability of the “even” outcome (which is payoff equivalent to “reciprocate” in Game 1). Participants again make decisions using the strategy method so that a MAP_ri_, reflecting a participant’s willingness to bear risk, could be established.

Based upon the theoretical and empirical data of studies using the [2] style of betrayal aversion identification, comparing a group’s MAP_ti_ to a groups MAP_ri_ provides a measure of the groups’ betrayal aversion. Since subjects in our task participate in both Game 1 and Game 2, we can obtain a measure of an individual subjects’ betrayal aversion. Eq 1 provides a continuous measure of the extent to which a participant is betrayal averse, BA_i_.

$$BA_{i}=MAP_{ti}-MAP_{ri}$$ (1)

If a participant places no utility weight on the knowledge of whether one’s social counterpart is reciprocal, BA_i_ should be equal to zero. A positive BA_i_ reflects that the agent requires a greater premium, as measured by the probability of the reciprocal outcome, to take the risk in the trust game compared to the risky dictator game. Thus a higher BA_i_ implies a greater level of betrayal aversion. On the other hand, a negative BA_i_ reflects that the agent requires a greater premium, as measured by the probability of the reciprocal outcome, in order to take the risk in Game 2 compared to Game 1. Such an agent could loosely be called betrayal seeking.

The precise classification of subjects as betrayal neutral, betrayal averse, or betrayal seeking is a choice that each researcher must address in their own experimental analysis. In the experimental analysis in the next section, in addition to using the continuous measurement of betrayal aversion, we bin subjects into the three categories in the following manner. We refer to subjects as betrayal neutral if BA_i_ is equal to zero, betrayal averse if BA_i_ is greater than zero, and betrayal seeking if BA_i_ is less than zero.

Before proceeding, we describe several important features of the BAET. First, note that the BAET integrates beneficial features of both the MAP procedure used initially in the [2] population level betrayal aversion identification design and beneficial features of the standard one-shot trust game procedure used in the population level betrayal aversion design in [14]. The dual MAP procedure we adopt provides a continuous measure of willingness to trust that a standard one shot game does not allow. Further, having subjects play both the trust version of the MAP trust game and the risky dictator version of the MAP game not only allows us to elicit an individual subject’s level of betrayal aversion, but additionally solves several of the problems of the aggregate level MAP procedure, pointed out in previous research [2,11,12,14 for more details). In particular, in the BAET design described here, all subjects know that the P_-I_ in both the risk and trust version of the MAP game come from their own pool of possible trustee counterparts and is by definition the same. Thus, expectations for what value P_-i_ takes is the same, making reference points in both games identical. Therefore loss aversion, which the previous studies indicated was a major concern with the MAP procedure, is no longer a factor potentially contributing to MAP differences. Similarly, since P_-i_ is generated from the same source, people face identical assessment costs when calculating it. Finally, since the same trustee decisions determine P_-i_ in both BAET games, an investor’s counterpart’s own contribution to the risk the counterpart will ultimately face is held constant. Note that a decision of a trustee to betray the trust of investor “i’s” trust decreases P_-i_, thus decreasing the probability that the investor “i”‘s MAP in either game is exceeded by P_-i_.

#### Risk Aversion

We assessed risk preferences using a modification of the Eckel and Grossman (henceforth EG, [20]) task with 7 gamble choices, each with a 50% chance of a high payoff and a 50% chance of a low payoff. The major differences in our version of the task are that there are seven gambles instead of the normal five, there was no riskless gamble, and no gamble which increases variance with no increase in expected value. Table 1 details the gambles and associated constant relative risk aversion (CRRA) parameter value intervals. Note in our version of the task, an increase in risk tolerance of one gamble choice is associated with a CRRA risk parameter increase of 0.45.

**Table 1:** Risk Choices and Associated Risk Preferences.

Gamble	Low Outcome	High Outcome	Expected Value	Implied CRRA Range
1	$12.24	$16.53	14.38	2.2 < r < ∞
2	$11.83	$17.40	14.62	1.75 < r < 2.2
3	$11.42	$18.27	14.84	1.3 < r < 1.75
4	$10.97	$19.14	15.06	0.85 < r < 1.3
5	$10.45	$20.01	15.23	0.4 < r < 0.85
6	$9.79	$20.88	15.34	-0.05 < r < 0.4
7	$8.89	$21.75	15.32	-∞ < r < -0.05

#### Laboratory Procedure

Fifty-six participants were recruited from sophomore level economics courses. Research was conducted at Virginia Tech’s Economic Research Lab and a session lasted approximately one and one half hours. When participants arrived at the lab, they completed a voluntary consent procedure approved by the University’s Institutional Review Board. Participants were then seated at computers equipped with privacy barriers where they completed the remainder of the experiment. Monitors insured that participants did not attempt to communicate or interact with each other or persons outside of the experiment at any point once seated at the computers.

Instructions for each phase of the experiment were computerized and read at the participants’ own pace immediately before completing that task. At the end of each section of the instructions, participants were asked to complete a quiz to check their understanding. Monitors were available to assist participants with any part of the instructions with which they had questions. The decision-making tasks and surveys were conducted using Z-tree [21]. (The Z-tree program used is available as a supplemental file online, in addition to a standalone software program for the betrayal aversion elicitation task (BAET).) A consistent conversion rate of $1 for every 30 lab points was announced at the beginning of the experiments.

Participants were all subjects who had previously participated in an iterated version of a trust exchange game similar to that of [22] in both the investor and trustee roles. Subjects observed their counterpart’s action and the resulting payoffs immediately after each decision. As such, we are confident that all subjects understood the nature of the trust game and the nature of reciprocation and betrayal that comes with the game.

Participants were informed that they had been randomly matched with a counterpart and then completed the betrayal aversion elicitation task described in the previous section. Participants made three decisions as part of the BAET, and were told that one of these decisions would be randomly chosen to determine their payment and the payment of a randomly matched anonymous counterpart.

Prior to learning the outcome of the BAET, participants completed the EG lottery selection risk task. The computer then randomly generated the outcome of the selected lottery, and summarized payment results of the BAET (as well as which task was randomly selected to be played) and lottery outcome. Participants were paid privately, in cash, for their participation after completing a series of post-task surveys. Payment was equal to experimental points earned in the tasks plus a $5 show-up fee and $5 for completing survey instruments.

## Results

Table 2 summarizes the subject level results of the betrayal aversion elicitation task. In the BAET, we observe that 23.2% of participants treat the trust game and risky dictator game the same, with *MAP*_*ti*_ = *MAP*_*ri*_, and are thus betrayal neutral. 44.6% of participants require a premium to trust, *MAP*_*ti*_ > *MAP*_*ri*_, and are thus betrayal averse. This 44.6% we identify as betrayal averse is very much in line with the 46.2% of investors that choose to avoid the knowledge of a personal betrayal in the “OPTION” treatment in [14] (two-tailed t-test, p = 0.817). The remaining 32.1% of participants require a premium to take the risk in the risky dictator game, *MAP*_*ti*_ < *MAP*_*ri*_, and are thus classified as betrayal seekers.

**Table 2:** Betrayal Aversion Summary Statistics (averages).

Group	Percent of Population	MAPti	MAPri	Trust Premium (MAPti-MAPri)
Betrayal Neutral MAPti = MAPri	23.2%	45.4%	45.4%	0.0%
Betrayal Averse MAPti > MAPri	44.6%	65.4%	41.4%	24.0%
Betrayal Seekers MAPti < MAPri	32.1%	42.8%	63.3%	-20.6%
All	100.0%	53.5%	49.4%	4.1%

**Fig 2:**
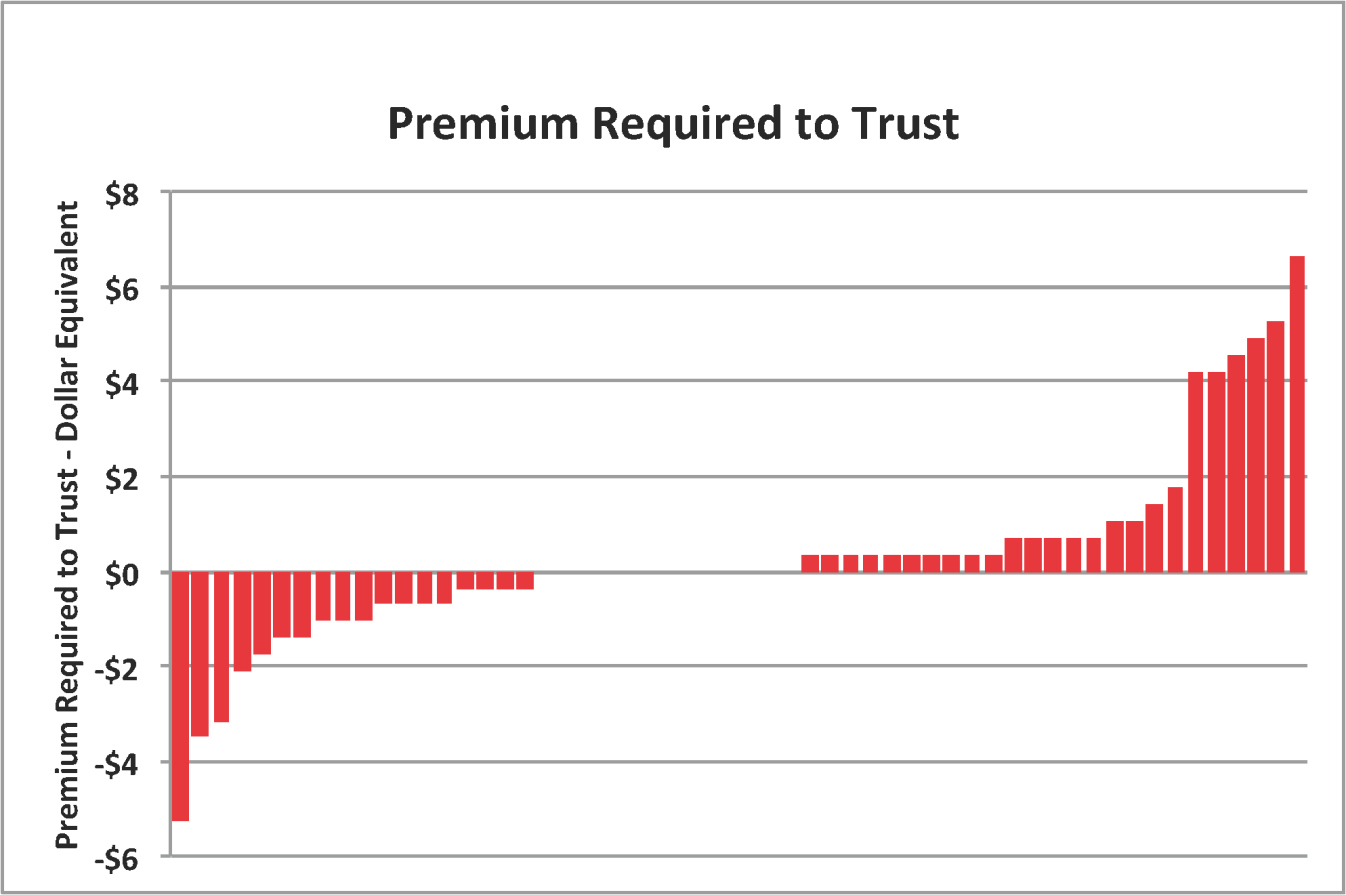
The figure illustrates the monetary premium each participant reported requiring in order for them to choose to trust a randomly matched trustee counterpart, ordered from the most Betrayal Seeking participant to the most Betrayal Averse. This premium is calculated as the extra probability required (or sacrificed) in order to trust, compared to the risk game (MAP difference), multiplied by the excess earnings that would result from the expected additional level of reciprocation.

Fig 2 summarizes the data in a different manner, showing the monetary premium required for each subject to be willing to engage in trust. This premium is calculated as the extra probability required (or sacrificed) in order to trust, compared to the risk game, multiplied by the excess earnings that the expected additional level of reciprocation would result in. Those subjects who require a premium to trust, e.g the betrayal averse subjects, on average require a $1.68 premium to trust, significantly greater than non-betrayal averse subjects (two-tailed Mann-Whitney, p<0.01). Those subjects who are willing to pay a premium in order to participate in the trust game on average would pay $1.44 to play the trust game over a risky dictator game. In the real world, this premium can be interpreted as the difference in monetary return required to induce an individual to allow an agent to invest their money for them.

Note that we measured betrayal aversion using subjects with prior experience with the trust game. The advantage of this is that we are confident that subjects understood the trust game underlying the BAET, however, there is also a chance that this experience might affect the data when compared with data collected with inexperienced subjects. If so, we suspect that in our data the apprehension over the negative emotions associated with discovering a betrayal would be lower, thus our measured betrayal aversion would be lower.

**Fig 3:**
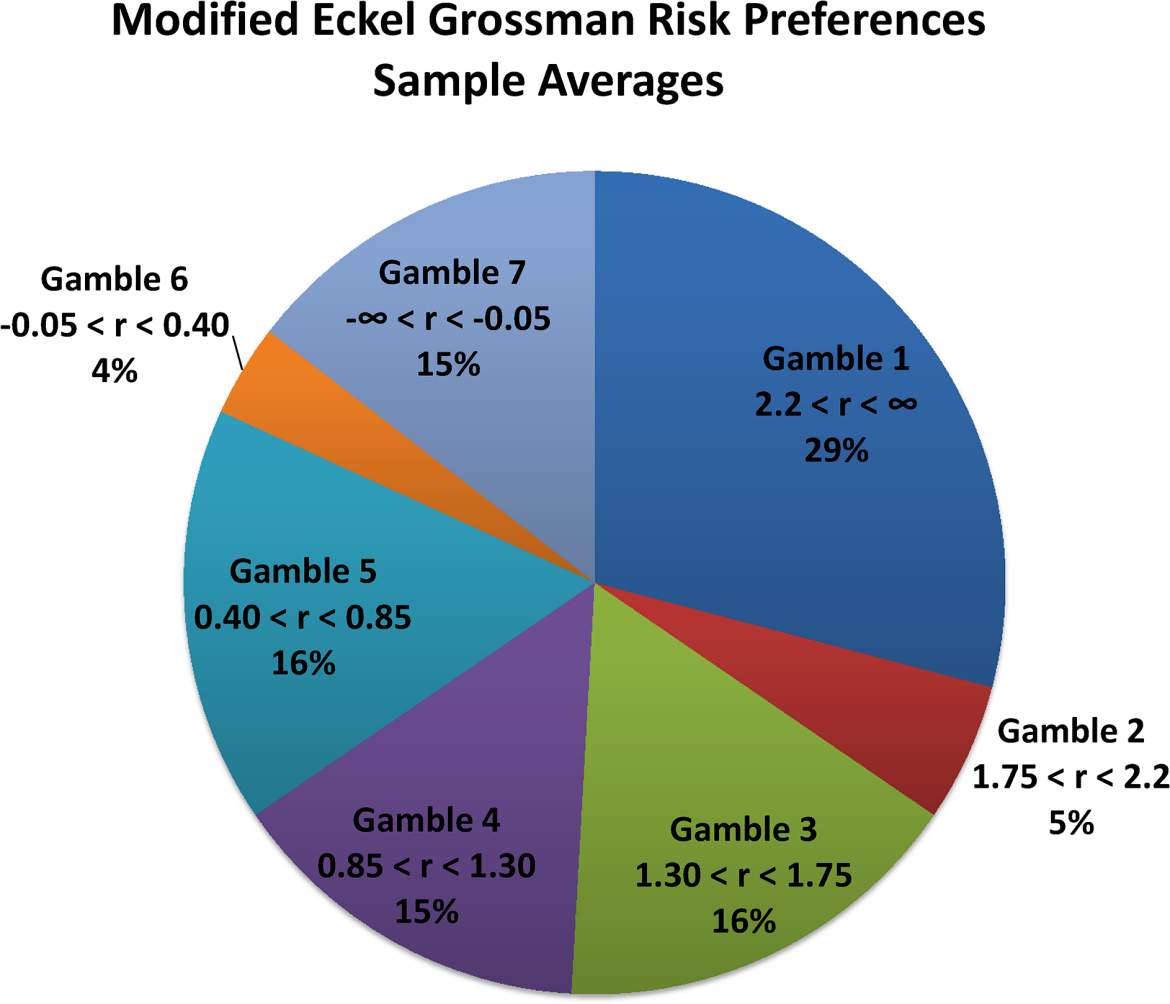
The figure reflects the proportion of participants falling into each risk preference interval from Table 1, based off of their Eckel Grossman risk task choice.

Fig 3, reports the distribution of participant decisions on the modified EG task in more detail. Participants choose 3.5 on average out of 7 in terms of gamble, associated with a CRRA coefficient of approximately 1.3. Fig 3 also demonstrates a benefit of expanding the number of gamble options in the EG task, eliminating the no-risk option and the increased risk without increased expected value option. We do not observe pooling in the middle of our seven-gamble version of the task (15% in option 4) as is often seen in standard implementation of the task (for example, [23] and [24] report 39.2% and 30.77% choosing option 3 in their 6 gamble versions of the task, respectively).

Turning to the relationship of individually measured betrayal aversion and risk aversion, we first examine the following hypothesis: that EG risk choices will show a stronger association with *MAP*_*ri*_ than *MAP*_*ti*_. As described in section 3.1 above, *MAP*_*ri*_ measured within risky dictator game reflects risk preferences for outcomes to oneself and a social counterpart that are determined by nature. To the extent that *MAP*_*ti*_ measured within the trust game differs from *MAP*_*ri*_, such differences must derive from differences in the source of risk; that is, a specific social counterpart rather than nature. (Note though, participants in the BAET know that group reciprocation probabilities determine the probabilistic “behavior” of nature.) Given that the preference measured in the EG derives from risk associated with nature rather than risk associated with the action of a social counterpart, we expect EG risk choices to be more strongly association with *MAP*_*ri*_ than *MAP*_*ti*_.

Indeed, we see a strong correlation between subjects’ EG gamble decision and their Game 2 (risky dictator game) *MAP*_*ri*_; that is, risk preferences revealed by the EG gamble decision are positively related to *MAP*_*ri*_ extracted from Game 2 (censored tobit, p<0.01). However, we see no analogous, significant relationship between risk preferences revealed by the EG gamble decision and the *MAP*_*ti*_ extracted from the Game 1 (trust game) decision (censored tobit, p = 0.349). A censored tobit including both MAPti and MAPti as independent variables shows the significant difference in these two measures ability to predict EG risk decisions (p = 0.013). Given the identical structure of the BAET components, the combination of positive and negative findings here confirms that *MAP*_*ri*_ is indeed related to one’s willingness to bear risk, while *MAP*_*ti*_ is associated with a second and unrelated factor deriving from the source of risk in the trust game. Further, the sensitivity of the risk component of the BAET (Game 2) to the EG metric, which itself has been related to individual differences [24] suggests that the *MAP* procedure here yields similarly robust metrics at the individual-level.

**Fig 4:**
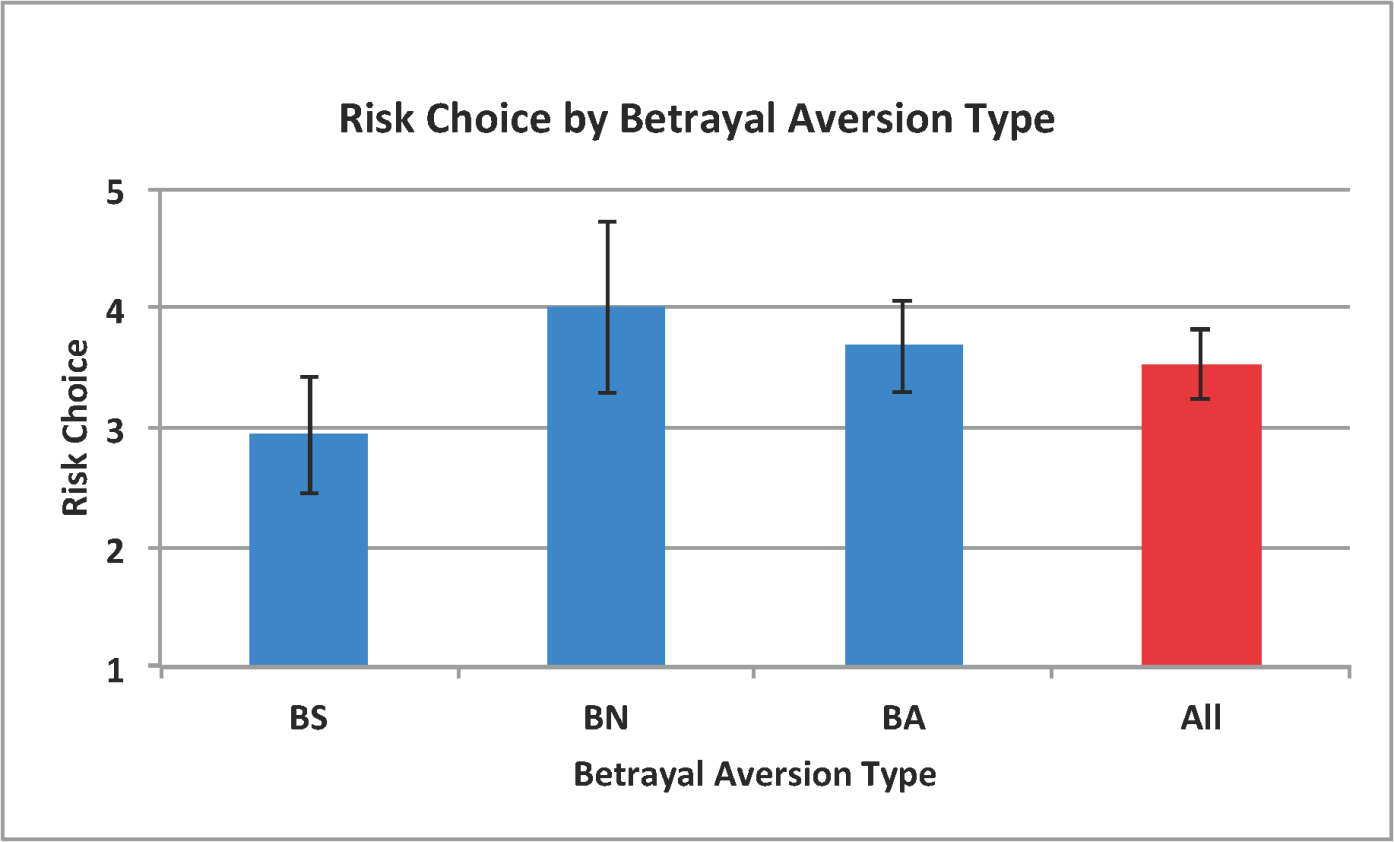
Bars reflect average risk choice (and standard error) by betrayal aversion type. Betrayal Seekers (BS) are those participants who required a premium to take the risk when nature chose the outcome (MAPti < MAPri). Betrayal Averse (BA) participants are those who required a premium to trust when their trustee chose the outcome (MAPti > MAPri). Betrayal Neutral (BN) participants are those who reported identical willingness to take risk whether nature or a trustee chose the outcome (MAPti = MAPri).

Examining the relationship between BA and EG, we observe, in our sample, that knowing one is betrayal averse reveals nothing about EG risk choice (two-tailed Mann-Whitney, p = 0.518; or OLS, p = 0.634) just as knowing ones EG risk preferences cannot tell us whether a subject is betrayal averse (logit, p = 0.619). Fig 4 illustrates these results in more detail by plotting subjects risk choices against their betrayal aversion type.

Finally, as a robustness check of the BAET for decisions framed in the loss domain, we ran a series of follow-up sessions (n = 71) where, in addition to surveys, participants completed both a standard BAET and then a second BAET where all the payments have 25 subtracted from them. In other words, the no-trust option becomes for both to lose 15, whereas the trustee chooses between both losing 10 and the investor loosing 17 and the trustee losing only 3. A signrank test shows no difference (two-tailed p = .31) between one’s betrayal aversion level in the loss and gain domain BAET task versions. Likewise we see a significant positive correlation between ones BA level in the gain domain and their BA level in the loss domain (OLS, p <0.001).

## Conclusions & Discussion

The major contribution of this work lies in the development of an elicitation procedure that provides a metric of betrayal aversion that can be assessed within an individual. While extensive previous work [20,25] has established robust measures of risk preferences previous work on betrayal aversion has focused on exploring the aggregate effects of betrayal aversion across populations, rather than within an individual. The modification we make to the designs of [2] and [14] provides a valuable tool with which to explore the influence of betrayal expectation across a host of environments where the research question requires a subject level measure or identification of betrayal aversion.

Here, we provide initial evidence that the new elicitation procedure provides measurements of betrayal aversion in individuals that are consistent with aggregate levels of betrayal aversion measured in past studies. Further, we show that the individual components of the BAET are more (risky dictator game) and less (trust game) associated with a standard measure of risk aversion, as would be expected of the procedure. Further, given that the two components of the BAET are identical, excepting the social versus nature source or risk, we have confidence that the BAET is measuring the same construct as group-level measures of betrayal aversion reported in previous studies [2, 3,11,12, 14,15].

Notably, we found that measures for betrayal aversion framed in the loss domain were similar to measures of betrayal aversion framed in the gain domain. In prior work using risk and trust games, studies have exclusively been carried out in the gain domain (i.e., where the betrayal reduces a potential gain). This differs from work in the consumer and legal decision-making, in which betrayals are typically framed as losses (i.e., the consumer or victim experiences a loss). Our findings, indicating consistent levels of betrayal aversion across domains, both suggests (i) that the BAET procedures may be useful for broader decision-making contexts (e.g., consumer and legal decision-making), and (ii) that betrayal aversion differs from risk aversion, in its sensitivity to gain and loss domains.

Our findings provide the first within subject evidence that betrayal aversion and risk preferences are distinct and uncorrelated factors that influence decision-making. We note that researchers have not controlled for betrayal aversion in research environments where there is both monetary risk and emotional risk. Our data suggests that in this type of environment an individual who is betrayal averse will appear to be somewhat less willing to take risk even if they have low monetary risk aversion. In these environments, therefore, models of behavior are likely to suffer from omitted variable bias or noise that makes attributing behavior to monetary risk preferences difficult. We suggest using the BAET to control for betrayal aversion in these contexts.

Future work may find the betrayal aversion elicitation task valuable in exploring research questions in many environments, from social and psychological settings to economic and political settings. Depending on the question of interest, it may be useful to establish the psychometric properties of the BAET and characterize to what extent previous experience with trust games and economic elicitations more generally may influence the magnitude of betrayal aversion assessed with this measure. Those interested in betrayal aversion in impersonal situations, such as with safety devices [1,6], may find the ability to control for participants’ betrayal aversion valuable using a modified version of our BAET that compares neutral safety products to safety products that have a positive probability of “betraying” the product user. Likewise the task could be easily modified to explore 3^rd^-party aversion to the betrayal of others and many other specific types of betrayal aversion.
